# The Antibody-Secreting Cell Response to Infection: Kinetics and Clinical Applications

**DOI:** 10.3389/fimmu.2017.00630

**Published:** 2017-06-01

**Authors:** Michael J. Carter, Ruth M. Mitchell, Patrick M. Meyer Sauteur, Dominic F. Kelly, Johannes Trück

**Affiliations:** ^1^Oxford Vaccine Group, Department of Paediatrics, University of Oxford, NIHR Oxford Biomedical Research Centre, Oxford, United Kingdom; ^2^University Children’s Hospital, Zurich, Switzerland

**Keywords:** B cells, antibody-secreting cells, plasmablasts, adaptive immunity, diagnosis, transcriptomics, monoclonal antibodies, B cell receptor sequencing

## Abstract

Despite the availability of advances in molecular diagnostic testing for infectious disease, there is still a need for tools that advance clinical care and public health. Current methods focus on pathogen detection with unprecedented precision, but often lack specificity. In contrast, the host immune response is highly specific for the infecting pathogen. Serological studies are rarely helpful in clinical settings, as they require acute and convalescent antibody testing. However, the B cell response is much more rapid and short-lived, making it an optimal target for determining disease aetiology in patients with infections. The performance of tests that aim to detect circulating antigen-specific antibody-secreting cells (ASCs) has previously been unclear. Test performance is reliant on detecting the presence of ASCs in the peripheral blood. As such, the kinetics of the ASC response to infection, the antigen specificity of the ASC response, and the methods of ASC detection are all critical. In this review, we summarize previous studies that have used techniques to enumerate ASCs during infection. We describe the emergence, peak, and waning of these cells in peripheral blood during infection with a number of bacterial and viral pathogens, as well as malaria infection. We find that the timing of antigen-specific ASC appearance and disappearance is highly conserved across pathogens, with a peak response between day 7 and day 8 of illness and largely absent following day 14 since onset of symptoms. Data show a sensitivity of ~90% and specificity >80% for pathogen detection using ASC-based methods. Overall, the summarised work indicates that ASC-based methods may be very sensitive and highly specific for determining the etiology of infection and have some advantages over current methods. Important areas of research remain, including more accurate definition of the timing of the ASC response to infection, the biological mechanisms underlying variability in its magnitude and the evolution and the B cell receptor in response to immune challenge. Nonetheless, there is potential of the ASC response to infection to be exploited as the basis for novel diagnostic tests to inform clinical care and public health priorities.

## Introduction

**Figure 1:**
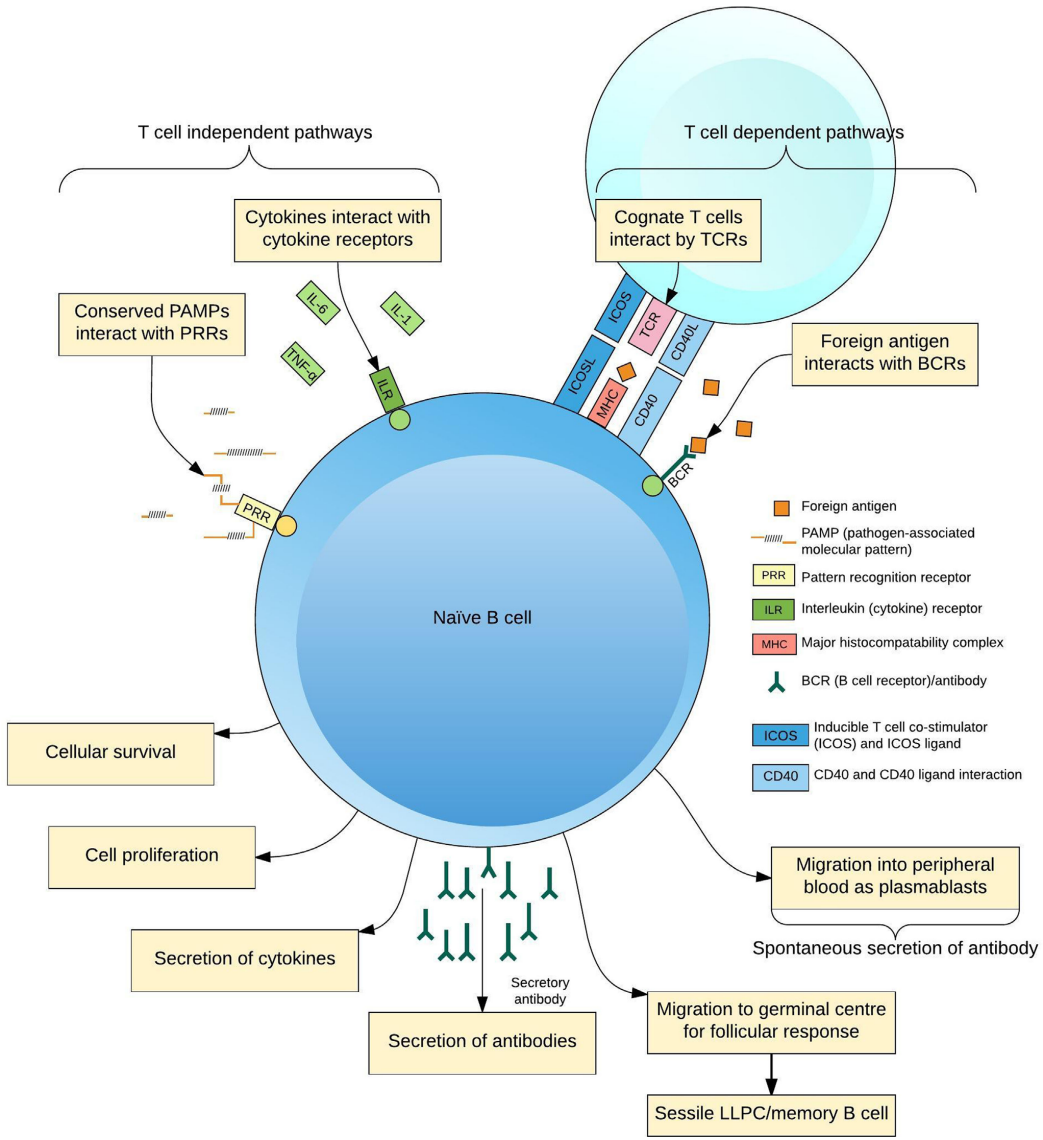
**Differentiation of B cells in response to antigen**. B cells receive activating signals by at least two pathogen non-specific mechanisms, pattern-recognition receptors (PRRs), and the cytokine context of the immune response; and two antigen-specific mechanisms, interactions with cognate T cells *via* the T cell receptor (TCR) and other ligands and B cell receptors (BCR)–antigen interaction. Integration of these signals predisposes the fate of the maturing B cell to a certain phenotype for the lifespan of the cell, simplistically resulting in plasmablasts, long-lived plasma cells (LLPCs), and memory B cells.

The production of pathogen-specific antibody by B lymphocytes (B cells) is of key importance for protection from infection. Naive B cells are activated through interaction with foreign antigen, cognate T cell receptors (TCRs) (1), pattern-recognition receptors (2), and cytokines (3) to form antigen-specific antibody-secreting cells (ASCs; plasmablasts and plasma cells), memory B cells and other subsets (4, 5) (Figure 1). Increasing insights into the cellular aspects of humoral immunity are emerging through a focus on plasmablasts. These are ASCs produced following antigen challenge that transiently circulate through peripheral blood before migrating to secondary lymphoid organs or bone marrow, or undergoing apoptosis. Plasmablasts thus represent an accessible and measurable subset of ASCs only detectable during an acute immune response.

Measurements of plasmablasts have been used extensively to describe the humoral immune response following vaccination (6). Measurements of plasmablasts may also provide data to inform the humoral response to infection and may translate into novel diagnostic tests. The development of novel diagnostic tests based on the plasmablast response to infection may circumvent difficulties with culture of organisms (usually lack of sensitivity due to difficulty accessing tissue and prior antibiotic administration), serological testing (usually lack of specificity unless a convalescent sample is also taken), or PCR-based methods (often lack of specificity, excepting testing of CSF). Additionally, the B cell receptors (BCRs) of plasmablasts are membrane-bound antibodies. As such, these BCRs could be used to inform vaccine development or to develop therapeutic monoclonal antibodies (7). Such therapeutic antibodies may be particularly important in the context of emerging infections or increasing antimicrobial resistance.

Antibody specificity and isotype and timing of the plasmablast response to infection are key to accurate measurements. However, these data have not previously been systematically reviewed. In this review we describe the data that exist on the timing and magnitude (kinetics) of the antigen-specific B cell responses following infection in humans. Our focus on ASCs (or where possible, the more specific subset of plasmablasts) reflects their importance in the production of antibodies, their dynamic numbers, and their transience in the peripheral blood: aspects of fundamental importance to their clinical applications. Where generated, memory B cells tend to persist at a low frequency; as so, they are not our focus, but have been recently reviewed in detail (8).

Search strategy. We searched MEDLINE with combinations of the following terms “B cell,” “plasmablast,” “plasma cell,” “antibody-secreting cell” AND “infection” AND “kinetics,” “dynamics.” We identified peer-reviewed articles of interest, and conducted further searches for terms identified as important (e.g. “antibodies lymphocyte supernatant,” “ELISpot,” “B cell receptor sequencing” in the context of infection).

## Techniques for Measuring Antigen-Specific B Cell Responses

### Challenges of Measuring B Cell Responses to Infection in Humans

Comparison of the ASC response to natural infection across studies is limited by varying definitions of ASC subsets. Here we use the term ASC to include all ASCs as defined by *ex vivo* assays such as enzyme-linked immunospot assay (ELISpot; below). We use the term plasmablast for ASCs that depict recognized cell surface markers for this B cell subset following immunophenotyping (9). We have eschewed the term “plasma cells” for clarity, since we will not discuss (usually sessile) long-lived plasma cells further. In general, numbers of ASCs are described as number per unit of blood, as a proportion of peripheral blood mononuclear cells (PBMCs), or as a proportion of peripheral blood B cells, as dictated by the techniques available to research teams (10). Antigen-specific ASCs may be further defined as a proportion of total (isotype-specific) ASCs (7), with varying definitions of antigen specificity.

### Techniques for Counting Specific Subsets of Cells

Fluorescence-based (flow) cytometry distinguishes immunophenotypes of cells by the binding of fluorescing monoclonal antibodies to defined cell surface markers. Thus, flow cytometry allows investigators to sort plasmablasts from other PBMCs. Markers of recent proliferation may be used to enrich samples for acutely proliferated plasmablasts, which may enhance specificity for the etiological diagnosis of acute infection (11). Replicability of experiments over time and between laboratories is paramount. As such, optimized panels of reagents for identifying specific populations and automated gating strategies have been developed (12).

ELISpot identifies subsets of cells by the binding of antibody to a chosen membrane-bound antigen. The addition of a substrate causes a color change where bound antibody is present, with the appearance of a spot corresponding to a single antigen-specific ASC (13). ELISpot is thus a highly sensitive technique because individual cells can be easily identified and counted. ELISpot is adaptable and can be applied to populations of cells that are either sorted by flow cytometry, or PBMCs separated from whole blood using density-dependent centrifugation. Although here we describe the detection of antigen-specific ASCs by the use of ELISpot and assay of antibody from lymphocyte supernatant (ALS) (Table 1), ELISpot has also been used for the immunophenotyping of B cell subsets for a variety of non-immunoglobulin markers (14). Unlike flow cytometry, ELISpot is a robust technique and can be used in laboratories in a variety of settings (10). A constitutive limitation to ELISpot, is the need for either PBMCs that have been recently sampled, or PBMCs that have been cryopreserved (15). Since antigen-specific ASCs form only a small proportion of PBMCs, even during the peak of an immune response, the number of antigen-specific ASCs limits the number of antigens that can be assayed. In addition, if fresh PBMCs are used, ELISpot plates must be prepared with predetermined antigens.

**Table 1:** **Studies investigating the use of ELISpot and ALS for the diagnosis of infection**.

Disease/pathogen	Setting	Methods and study patients/volunteers	Results	Reference
Enterotoxigenic *Escherichia coli* dysentery	USA (challenge study)	20 adult volunteers with live oral attenuated ETEC vaccine; 20 adult volunteers with ETEC oral challenge	ALS against *E. coli* antigens IgA: positive in 75–95% of infected subjects. ELISpot: positive in 85–100% of infected subjects (depending on antigen measured)	Carpenter et al. (16)
		Comparison of ELISpot assays for IgA ASCs and IgA ALS (for IgA) at day 0, day 7, and day 21 following challenge	ALS against CFAII IgA: positive in 75% of vaccinees; ELISpot: positive in 90% of infected subjects	
		Antigens for ALS ELISA, time-resolved fluorescence antibody assay, and ELISpot: CFA/II (vaccinees); LT, CS3, CS1 (infected subjects)	Both ALS and ELISpot considerably higher than fecal IgA measurements against matched antigens	

	Urban Bangladesh	46 adult and child patients with CS6-expressing ETEC diarrhea, 10 local adult \controls, and 10 local child controls	ALS against CS6: 89% with positive with IgA, 100% positive with IgG. ELISpot against CS6: 100% positive with IgA (GMC 430 ASC/million PBMC), 75% positive with IgG.	Qadri et al. (17)
		Comparison of ELISpot assays for ASCs on 12 adult patients and ALS for all 46 patients, at day 2 and day 7, versus ELISpot and ALS on healthy controls. Antigens for ALS and ELISpot: CS6 and CS5 + CS6		
			No responses detectable by ALS or ELISpot in healthy controls	

*Shigella* spp. dysentery	USA (challenge study)	30 adult volunteers with oral live attenuated *S. dysenteriae* type 1a vaccine; 10 adult volunteers with *S. flexneri* 2a oral challenge	ALS for *Shigella*-LPS IgA ASCs: sensitivity of 79%, specificity of 75% versus ELISpot. ALS for *Shigella*-LPS IgA ASCs: sensitivity of 79%, specificity of 75% versus ELISpot. Details on timing or magnitude of ASC responses by ELISpot of ALS not given.	Feller et al. (18)
		Comparison of ELISpot assays for ASCs and ALS (for IgA and IgG). No details of time points		
		Antigens for ELISA of ALS and ELISpot: *Shigella*-LPS		

Cholera	Urban Bangladesh	30 adult patients with acute cholera and 10 local healthy controls	Peak cholera-specific ASCs at day 7 (median peak CtxB-specific IgA ASCs ~ 350/million PBMCs, LPS-specific IgA ASCs ~900/million PBMCs)	Qadri et al. (19)
		Comparison of ELISpot assays for IgA ASCs and IgA ALS at presentation to hospital and day 7. Antigens for ALS and ELISpot: CTxB, cholera LPS and MSHA	ALS for IgA CtxB-specific ASCs: sensitivity of 94% versus vibriocidal serum response. ELISpot for IgA LPS-specific ASCs: sensitivity of 94% versus vibriocidal serum response	

	Urban Bangladesh	17 children 3–5 years of age, 17 older children and 68 adults with acute cholera.	Similar antibody/ASC responses across age groups. Returned to baseline by day 30	Leung et al. (20)
		Comparison of vibrocidal antibodies, antigen-specific antibodies, ELISpot for IgM/A/G ASCs and (stimulated) memory B cells at day 2, day 7, and day 30	Peak cholera-specific ASCs at day 7 (median peak CtxB-specific IgA ASCs ~100–200/million PBMCs, LPS-specific IgA ASCs ~200–400/million PBMCs)	

	Urban Bangladesh	Nine adult patients with acute cholera infection, eight adults vaccinated with oral cholera vaccine (Dukoral)	Median peak CtxB-specific IgG ASCs at ~370/million PBMCs (patients) and ~200/million PBMCs (vaccinees)	Rahman et al. (21)
		Comparison of ASC responses between infection and post-vaccination using ELISpot (antigens: CtxB and cholera LPS) on days 2, 7, and 30 (infection) and days 0, 7, and 21 (vaccination)	Detectable CtxB/LPS-specific IgA/IgG ASC responses in 9/9 patients and 5/8 post-vaccination at day 7 (returned to baseline during convalescence)	

Enteric fever	Urban Bangladesh	112 adult and child patients with fever of unknown etiology of 3–7 days duration	IgA ALS against LPS and whole-cell preparation: sensitivity 88% versus blood culture	Sheikh et al. (22)
		Comparison of ALS and plasma antibodies versus comparator standards (classic clinical presentation, Widal titer, blood culture) at day 0, day 5, and day 20 following presentation	IgA ALS and plasma antibodies across comparator groups supports possibility of ALS detecting blood culture-negative enteric fever	
		Antigens for IgA and IgA ALS: typhoid LPS, whole-cell preparation, and membrane preparation (MP)		

	Rural Bangladesh	243 adult and child patients with clinically suspected enteric fever (39 blood culture positive), 32 febrile (non-enteric fever) controls (both groups with fever of 3–7 days duration), and 74 local healthy controls	Sensitivity of 100% for blood culture positive enteric fever, specificity of 78–97% (depending on definition of a true-negative case)	Khanam et al. (23)
		ALS at presentation to hospital/enrollment (all patients/controls) and day 7 and day 21 in 38 patients. Antigens for IgA ALS: typhoid MP		

Tuberculosis	Urban Bangladesh	49 adult patients with smear-positive pulmonary TB; 35 adult patients with other pulmonary diseases; 35 healthy local controls	IgG ALS against BCG: sensitivity of 93% and specificity of 80% to detect smear-positive in pulmonary TB	Raqib et al. (24)
		ALS at presentation to hospital only. Antigens for IgG ALS: BCG and PPD		

	Urban Bangladesh	58 child patients with clinical TB; 16 child patients with other infections; 58 healthy child controls	Of patients with clinical TB: 15% positive by culture of sputum/gastric lavage, 64% positive by published clinical scoring charts, 91% positive by ALS	Raqib et al. (25)
		ALS for all patients/controls (and standard microbiology for patients) at day 1, day 60, and day 180 following presentation. Antigens for ALS: BCG	All children with TB had significantly higher ALS titers that non-TB patients or controls. Declining ALS titers in children with active disease following treatment	

	Urban Bangladesh	212 adult patients with suspected TB; 100 adult patients with other diseases; 25 healthy adult controls	Sensitivity of 91%, specificity of 88% specificity using BCG-specific IgG ALS for pulmonary TB (smear/culture positive) versus non-TB patients	Rekha et al. (26)
		ALS at presentation to hospital only. Antigens for ALS: BCG and other *M. tuberculosi*s antigens		

	Urban Ethiopia	84 adult patients with smear-negative symptomatic TB (pulmonary, pleural, and lymph node); 12 adult patients with other diseases; 45 adult patients with latent TB; 40 non-TB controls	BCG-specific plasmablasts median of 6% of plasmablasts in symptomatic TB versus 3% in latent TB (*p* < 0.001)	Ashenafi et al. (27)
		Comparison of IgG ALS, BCG-specific plasmablast enumeration (flow cytometry), Quantiferon Gold, tuberculin skin test. Antigens for IgG ALS: BCG	ALS: 86–90% sensitivity, 80% specificity for symptomatic TB versus latent TB. ALS: 86–91% sensitivity, 94% specificity for symptomatic TB versus non-TB controls	

	Urban Bangladesh	224 patients <5 years of age with severe malnutrition and radiological features of pneumonia/pulmonary infection. 15 patients with microbiologically confirmed TB; 41 patients with non-confirmed TB; 168 patients not TB	Similar proportions of “positive,” “bordeline” and “negative” ALS results between three groups (confirmed TB, non-confirmed TB, not TB)	Chisti et al. (28)
		ALS at presentation to hospital. Antigens for IgG ALS: BCG	Sensitivity of 67% and specificity of 51% for confirmed TB versus not TB.	

	Urban China	62 adult patients with culture-positive pulmonary TB; 53 adult patients with other pulmonary diseases; 47 healthy adult controls	ALS: 97% sensitivity, 85% specificity for culture-positive TB versus healthy controls. ALS: 68% sensitivity and 93% specificity for culture-positive	Jiao et al. (29)
		IgG ALS at presentation to hospital only. Antigens for IgG ALS: *M. tuberculosis* acid phosphatase	TB versus patients with other pulmonary diseases	

Pneumococcus and other bacterial respiratory pathogens	Finland	16 adult patients with bacteremic pneumococcal pneumonia and 14 healthy controls	All patients had pneumococcal-specific IgG ASCs ≥10/million PBMCs (range 10–1,000/million); all controls had 0–1/million PBMCs detected: sensitivity and specificity of 100% in this small retrospective case series	Palkola et al. (30)
		ELISpot to detect pneumococcal-specific IgM/A/G ASCs on day 7 following bacteremia or from healthy controls. Antigens for ELISpot: patient’s pathogen, or mixed capsular polysaccharides (controls)		

	Finland	24 adult patients with acute sinusitis of acute tonsillitis and 9 adult healthy controls	All patients had pathogen-specific IgG ASCs ≥10/million PBMCs (range 10–1,000/million PBMCs); controls had no pathogen-specific ASCs detected: sensitivity and specificity of 100% in this case series	Palkola et al. (31)
		*Streptococcus pneumoniae, H. influenza*, and β-hemolytic streptococci isolated from patient swabs		
		Patient blood samples taken 7–14 days following onset of symptoms		
		ELISpot to detect antigen-specific IgM/A/G ASCs patient’s pathogen and pathogen panel for controls		

UTI caused by *E. coli* and other bacteria	Finland	10 adult patients with lower UTI and 17 patients with pyelonephritis due to *E. coli, Enterococcus* spp., *Enterobacter* spp., and staphylococci	Pathogen-specific IgA ASCs in 100% of patients with pyelonephritis (mean 787, 95% CI 738–836/million PBMCs) and 70% of patients with lower UTI (mean 6 95% CI 6–12/million PBMCs). Lower responses for other Ig isotypes	Kantele et al. (32)
		Patient blood samples 7 days following onset of symptoms and in convalescence		
		ELISpot to detect antigen-specific IgM/A/G ASCs to patient’s own pathogen	Downward trend in ASCs at convalescence, but pathogen-specific ASCs still detectable at 7 weeks in some patients	

	Finland	14 adult patients with lower UTI and 11 patients with pyelonephritis due to *E. coli*. [Patients previously report in Kantele et al. (32)]	IgA ASC responses greater than IgG and IgM; and responses to whole cell greater than to P fimbria and OmpA. Mean P fimbria-specific IgA ASC 294/million PBMCs	Kantele et al. (33)
		Patient blood samples at 7 days following onset of symptoms	P fimbria-specific IgA ASC responses in 100% of patients with P fimbria-positive *E. coli* pyelonephritis, and 20% of those with P fimbria lower UTI	
		ELISpot to detect whole cell, P fimbria or OmpA-specific IgM/A/G ASCs		

	Finland	37 children with pyelonephritis due to *E. coli*	Mean IgM ASC 19 cells, IgA ASC 16 cells, IgG 6 cells/million PBMCs. IgM predominating in children <2 years of age	Kantele et al. (34)
		Patient blood samples 1–3 days following admission and 7 days following admission, and convalescence	33/37 (89%) children with detectable pathogen-specific ASCs. Responses correlated with increasing age, with four non-responders <1 year of age	
		ELISpot to detect IgM/A/G ASC to patient’s own pathogen and P fimbria		

Influenza	UK (challenge study)	12 healthy adult volunteers	Median Br59-specific IgG ASCs 166 (IQR 111–121)/million PBMCs at day 7. Antigen-specific ASCs were absent at other time points. Antigen-specific ASCs correlated with viral load and symptom score	Huang et al. (35)
		Blood samples at day −2, day 3, day 7, and day 28 relative to challenge		
		ELISpot to hemagglutinin to detect H1N1Br59-specific IgG ASCs		

RSV	UK (challenge study)	61 healthy adult volunteers	Infected volunteers showed median peak RSV-specific IgG ASCs of ~300/million PBMCs at day 10. Antigen-specific ASCs were undetectable prior to day 7 and after day 14. RSV-specific IgG ASCs were largely undetectable in on-infected volunteers	Habibi et al. (36)
		Blood samples at day 0, day 3, day 7, day 10, day 14, and day 28 relative to challenge		
		ELISpot to detect RSV-lysate-specific IgM/A/G ASCs		

	USA	40 adult patients with RSV infection	RSV-specific IgG ASCs detectable by day 2 of illness and detected in 90% of cases by day 11 (mean 200 RSV-specific IgG ASCs/million PBMCs). RSV-specific ASC responses were detectable in 48% of cases in convalescence (days 22–45)	Lee et al. (37)
		Blood samples at enrollment, days 10–16 and days 22–45 following onset of symptoms		
		ELISpot to detect RSV-F protein-specific IgG ASCs		

	USA	97 adult patients with respiratory virus infection in total; data presented on 11 patients with RSV and 11 patients with influenza infection; and healthy controls including 19 adults recently vaccinated with against influenza, tetanus, HPV or HBV	RSV-specific IgG ASCs detectable by day 2 of illness, and detected in 100% of cases by day 11 (median ~1,000 ASCs/million PBMCs) and absent in influenza infection and controls. Similarly, influenza-specific IgG ASCs detected in 100% of cases by days 4–11 (median peak ~1,000 ASCs/million PBMCs) and absent in RSV infection and controls	Lee et al. (38)
		Patient blood samples at days 4–11 following symptom onset (enrollment), post-vaccine samples 6–7 days following vaccination		
		ELISpot to detect RSV-F protein-specific IgG ASCs, influenza antigens and vaccine antigens (as above)		

Dengue	Urban Thailand	46 adult patients with severe dengue infection	Median peak dengue-specific IgG ASCs ~15,000/million PBMCs (range 10–150,000/million PBMCs). Lower responses with IgA and IgM ASCs. Dengue-virus specific ASCs absent from controls and in convalescence	Wrammert et al. (39)
		Patient blood samples on day of admission (median 6, range 2–8, days following onset of fever) and convalescence		
		ELISpot to detect dengue-virus-specific IgM/A/G ASCs (using purified dengue virions)		

	Brazil	84 child and adult patients with primary or secondary dengue infection, 15 controls with fever from other causes, and 10 local healthy controls	Median peak dengue-specific IgG ASCs ~20,000/million PBMCs (range 3,000–100,000/million PBMCs), encompassing ~50% of all IgG ASCs (to any antigen)	Garcia-Bates et al. (40)
		Patient blood samples on day of admission (median 6, range 1–9, days following onset of fever) and convalescence	Peak of IgG ASCs (to any antigen) between day 3.5 and day 7 following onset of symptoms and correlated with onset of symptoms	
		ELISpot to detect dengue-virus-specific IgG ASCs (using purified dengue virions)		

Mumps	USA (outbreak)	Seven adult with acute mumps infection; 16 adult volunteers vaccinated with mumps virus vaccine (following previous infection/vaccination)	Mumps virus-specific ASCs 10–100/million PBMCs from day 7 to day 14 (6 patients), and 1.5/million PBMCs on day 28 (1 patient)	Latner et al. (41)
		Patient blood samples between day 7 and day 28 of onset of symptoms. Volunteer blood samples at day 0, day 7, and day 28 and 35 weeks	Pre-vaccine and 35 weeks post-vaccine samples all mumps virus-specific ASCs <0.5/million PBMCs	
		ELISpot to detect IgG ASCs to purified mumps virions	100% sensitivity and specificity for diagnosis of acute mumps infection	

EV-71	Taiwan	28 children with acute EV-71 infection admitted to hospital	Mean EV-71-specific IgM ASCs 472, IgA ASCs 187, IgG ASCs 491/million PBMCs	Huang et al. (42)
		Blood samples on admission (mean 4.7 days following onset of symptoms) and convalescence	Responses largely undetectable from day 8 following onset of symptoms	
		ELISpot to detect IgM/A/G ASCs to purified EV-71 virions	IgM ASC response predominated in younger children	

Falciparum malaria	UK (challenge study)	Nine adult volunteers initially vaccinated with experimental merozoite (blood-stage) vaccine, subsequently exposed to malaria challenge infection	No detectable MSP1-specific IgG ASC responses at days 7, 11, and 35 in volunteers undergoing malaria challenge infection (despite merozoite parasitemia by day 7)	Elias et al. (43)
		Blood samples at day –1, day 7, day 11, and day 35 following malaria challenge infection	Possible questions regarding lack of later time points (since merozoite parasitemia, not prehepatic sporozoites, may act as immune stimulus)	
		ELISpot to detect MSP1-specific IgG ASCs		

*ALS, antibody from lymphocyte supernatant; ASCs, antibody-secreting cells; CtxB, cholera toxin B subunit; PBMCs, peripheral blood mononuclear cells; LPS, lipopolysaccharide; MSHA, mannose-sensitive hemagglutinin; TB, tuberculosis; BCG, Bacille Calmette-Guérin; PPD, purified protein derivative; GMC, geometric concentration; ETEC, Enterotoxigenic Escherichia coli; CFA/II, E. coli colonizing factor; CS1, CS3, CS6, coli surface antigens; LT, heat-labile E. coli toxin; UTI, urinary tract infection; OmpA, outer membrane protein A; RSV, respiratory syncytial virus; HPV, human papillomavirus; HBV, hepatitis B virus; EV-71, Enterovirus-71; MSP1, merozoite surface protein 1; MP, membrane preparation*.

### Other Techniques to Assess Antigen-Specific ASCs

Assay of ALS measures the sum of antibodies produced by ASCs during a period of *in vitro* cell culture. The method is simple: peripheral blood is sampled, and PBMCs are separated by density-dependent centrifugation, washed, and incubated for 24–48 h in cell culture media (44). The resulting supernatant is enriched for antibodies that were secreted during *ex vivo* incubation by recently activated ASCs. As with ELISpot, this technique has potential to be developed into a diagnostic test for the etiology of acute infections and has already been applied with some success to clinical tuberculosis (26, 27, 45) and enteric fever (22, 23), as well as vaccine responses (16) (Table 1). Although frozen cells can also be used for the ELISpot (15), ALS samples can be more easily frozen and transported to centralized laboratories, allowing responses from many antigens from multiple pathogens to be assayed. This may be of importance given progress in serological techniques (46) and the re-emphasized need for seroepidemiology in the context of emerging infectious diseases (47). Important questions regarding optimal timing of samples relative to onset of symptomatic infection and its sensitivity in comparison to ELISpot, remain.

At present, the use of ALS and ELISpot to detect antigen-specific ASCs for the diagnosis of infection is largely limited to research laboratories, with the exception of an established T cell ELISpot test for tuberculosis [T-spot.*TB* (48)]. This reflects the need for rigorous standardization of laboratory procedures to detect antigen-specific ASCs (or their secretions) prior to clinical trials or implementation into clinical practice. ALS and ELISpot also require techniques that, although simple, will be unfamiliar to the majority of microbiology technicians such as the separation and washing of PBMCs from fresh blood. ELISpot plates with predetermined antigens for use in low-technology settings (10), or the use of ALS (44), which can be transported relatively simply to reference laboratories for ELISA, have been adapted to these ends.

An alternative to measuring antibodies (and other proteins) produced in response to infection is to measure the upstream transcription of genes across the genome. As such, measurement of the abundance of RNA species in whole blood or PBMCs using gene expression microarrays have been used to define the transcriptome in response to vaccination or infection. Theoretically, this approach is “hypothesis-free” regarding genes that may be differentially regulated in response to immunological challenge and relatively unbiased regarding the detection of genes (49). Additionally, differential gene expression precedes translation of proteins, and therefore may define immune response to infection prior to the detection of antigen-specific ASCs or antibodies in blood. Such gene-level data from microarray experiments can accurately distinguish between bacterial and viral infections at time of presentation to hospital (50–52) and may potentially distinguish within groups of bacterial or viral infections. Gene-level data may also be used to investigate immunological pathways activated by vaccination or colonization/infection (53). However, microarrays are inherently unable to define the hypervariable genes encoding the heavy (*IGH*) and light (*IGL/IGK*) chains of the BCR, and do not inform on the antigen specificity of the ASC response. Thus RNA microarrays are unlikely to distinguish between closely related infections or between serotypes of the same infecting pathogen.

The recent development of next-generation sequencing has facilitated investigation of the ASC response to immunization and infection at a fundamental genetic level. Sequencing of genes encoding the BCR has resulted in the successful production of monoclonal antibodies to influenza (7). Further, convergence of sequences encoding the BCR has been described in populations of B cells in the blood of adults following vaccination (54–57). The clinical implications and challenges of these novel techniques are discussed below.

## ASC Kinetics

### Antigen-Specific ASC Responses to Vaccination

**Figure 2:**
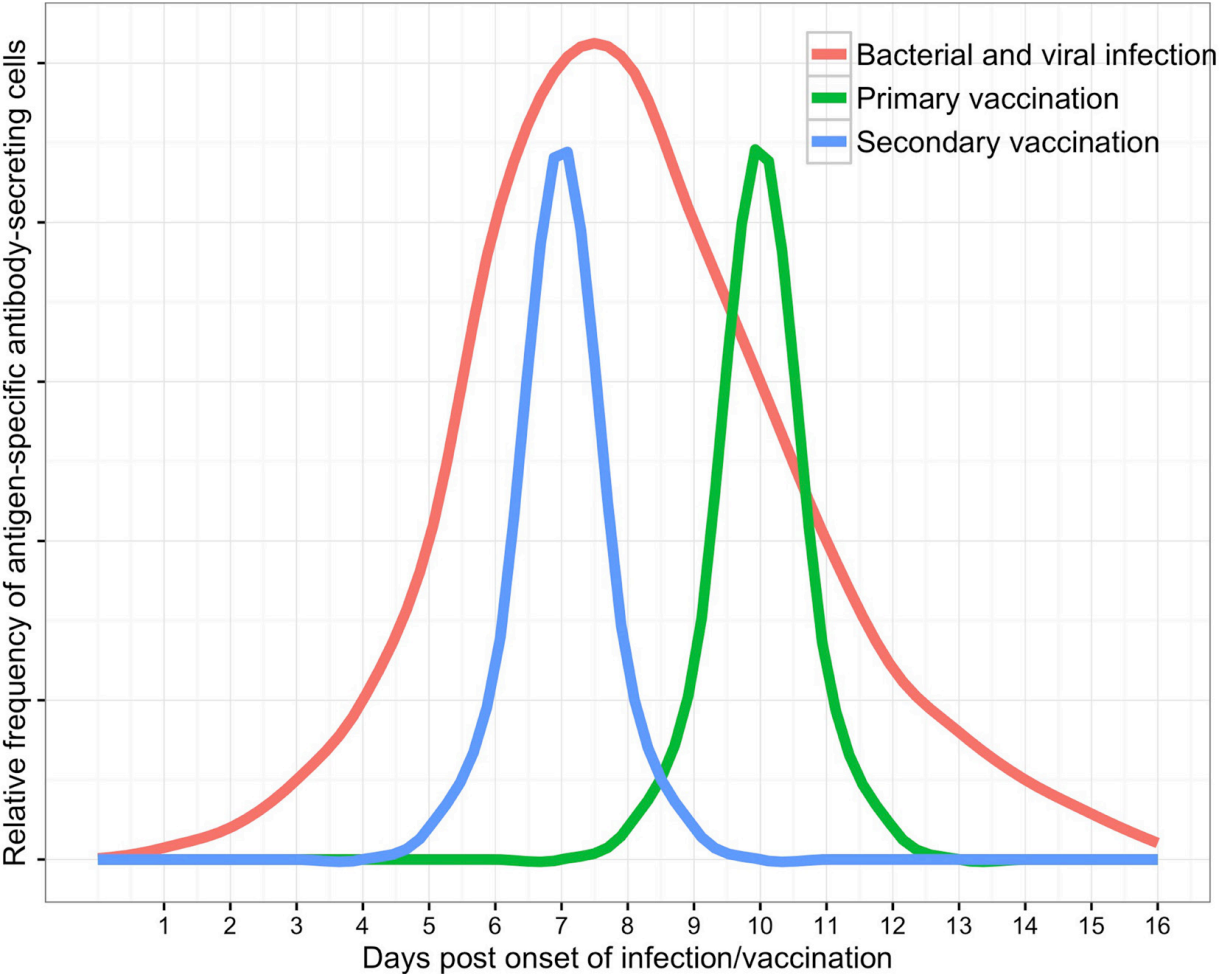
**Schematic of antibody-secreting cell responses to infection as measured in peripheral blood (with supporting data and variability discussed in the text)**.

B cell subsets in peripheral blood are dynamic populations. A detailed knowledge of ASC kinetics is therefore paramount when sampling blood to investigate the ASC response to infection (Figure 2). ASC responses to natural infection may be dependent upon host genetic factors (58, 59), the site of antigen presentation (mucosal surfaces, secondary lymphoid organs, or systemic circulation), the type or quantity of antigen to which the host is exposed, and integration of other immune signals (derived from T cells, pattern-recognition receptors, and cytokines). Onset of natural infection is typically difficult to define, particularly since mucosal colonization with a pathogenic organism is likely to be a necessary precursor to many diseases (60–62). Practical limitations on sampling blood from patients with infections have limited the temporal resolution of studies of the ASC response to infection. These challenges are reduced in vaccine studies (6), where a known measure of antigen is administered at a specific time. The broad themes emerging from detailed data on ASC responses to vaccines can therefore be used as a point of comparison with data from the ASC response to infection.

The timing of the ASC response to vaccination is consistent between most individuals. Vaccination of adult volunteers with polysaccharide–protein conjugate intramuscular (IM) vaccines induced peak antigen-specific ASC responses at 10 days following vaccination of non-primed individuals. In primed individuals, the peak ASC responses occurred earlier, at a median of 7 days following vaccination (6). These adult volunteers had a median frequency of antigen-specific ASCs of ~50% of all ASCs in peripheral blood at peak time points (13, 63–65). Similarly, vaccination with protein IM vaccines induced peak antigen-specific ASC responses at 6–7 days following vaccination in primed adult volunteers. However, the magnitude of IgG antigen-specific responses varied considerably between immunoglobulin subclasses, and between antigens and were highest against tetanus toxoid (66, 67), followed by pertactin (pertussis antigen) (66) and hemagglutinin (influenza antigen) and lowest for diphtheria toxoid (63) and hepatitis B surface protein (56). Moreover, responses varied between individuals in most studies by an order of magnitude.

The timing and magnitude of the ASC response to mucosal vaccines are similar to that induced by IM vaccines and appear conserved between bacterial and viral antigens. Antigen-specific ASC responses to killed oral cholera vaccine (OCV, trade name Dukoral) were measurable in peripheral blood at days 6–7 but not at day 3 or day 28 following vaccination of adults in Bangladesh (21). Similarly, antigen-specific ASC responses to live attenuated influenza vaccine were measurable in blood between day 7 and day 12 following intranasal administration to adults (68). Interestingly, both of these mucosal vaccines induced an antigen-specific IgG ASC response that was 2–3 times higher than the corresponding IgA ASC response, suggesting a systemic immune response to these localized mucosal vaccine challenges.

### Antigen-Specific B Cell Responses to Bacterial Antigens

#### ASC Responses to Infection in Comparison to Vaccination

The timing and magnitude of ASC responses may differ between vaccination (particularly with non-live vaccines) and infection. The details of studies of ASC responses to infection with bacteria, viruses, and malaria are described in Tables 1 and 2. Two studies have directly compared the ASC response to vaccination and infection: enterotoxigenic *Escherichia coli* (ETEC) vaccine and challenge infection and OCV and natural infection with *Vibrio cholerae*.

**Table 2:** **Detection of antigen-specific ASCs *via* ELISpot or ALS by disease/pathogen and time point of sampling relative to onset of symptoms**.

Disease	Setting	Optimum method	Detection of pathogen-specific ASCs by days following onset of symptoms																Reference
	Day		1	2	3	4	5	6	7	8	9	10	11	12	13	14	21	28+	
Enterotoxigenic *E. coli* dysentery	USA challenge study)	IgA ALS and ELISpot to LT-specific ASCs							+			+							Carpenter et al. (16)
	Urban Bangladesh	IgA ALS and ELISpot to CS6-specific ASCs		+/−					+++										Qadri et al. (17)
*Shigella* spp. dysentery	USA (challenge study)	IgA ALS and ELISpot to LPS-specific ASCs							No data regarding timing of ASC response										Feller et al. (18)
Cholera	Urban Bangladesh	IgA ALS and ELISpot to LPS-specific ASCs		+/−					+++++++++										Qadri et al. (19)
	Urban Bangladesh	IgG ELISpot to CtxB-specific ASCs		−					++									−	Leung et al. (20)
	Urban Bangladesh	IgG ELISpot to CtxB-specific ASCs		−					++++								−		Rahman et al. (21)
Enteric fever	Urban Bangladesh	IgA ALS to LPS			+	+	+	+	+	+	++	++	++	++			+	+	Sheikh et al. (22)
	Rural Bangladesh	IgA ALS to typhoid membrane preparation			+	+	+	+	+										Khanam et al. (23, 24)
Tuberculosis	Urban Bangladesh	IgG ALS to BCG	________________________________________________[+]________________________________________________																Raqib et al. (24)
	Urban Bangladesh	IgG ALS to BCG	________________________________________________[+]________________________________________________																Raqib et al. (25)
	Urban Bangladesh	IgG ALS to BCG	________________________________________________[+]________________________________________________																Rekha et al. (26)
	Urban Ethiopia	IgG ALS to BCG	________________________________________________[+]________________________________________________																Ashenafi et al. (27)
	Urban Bangladesh	IgG ALS to BCG	________________________________________________[+]________________________________________________																Chisti et al. (28)
	Urban China	IgG ALS to SapM	________________________________________________[+]________________________________________________																Jiao et al. (29)
Pneumococci and other respiratory bacteria	Finland	IgG ASCs to pathogen							++										Palkola et al. (30)
	Finland	IgG ASCs to pathogen					+		++		+	++ ++	+	++ +		++			Palkola et al. (31)
UTI caused by *E. coli* and other bacteria	Finland	ASCs to pathogen			+/−				+ (LUTI), +++++ (PN)									+/−	Kantele et al. (32)
	Finland	ASCs to P fimbria			+/−				+++++ (PN)									+/−	Kantele et al. (33)
	Finland	ASCs to pathogen			_________________+++++_______________														Kantele et al. (34)
Influenza	UK (challenge study)^a^	IgG ASCs to H1N1	−				++											−	Huang et al. (35)
	USA	IgG ASCs to various influenza antigens		___________________________________+++++___________________________________															Lee et al. (38)
RSV	UK (challenge study)^a^	ASCs to RSV lysate					−			++				+/−				−	Habibi et al. (36)
	USA	ASCs to RSV F protein		+/−	±	++	++	++	++	++	+++ ++	++	+				+/−	−	Lee et al. (37)
	USA	ASCs to RSV F protein		___________________________________+++++___________________________________															Lee et al. (38)
Dengue	Urban Thailand	IgG ASCs to dengue virions		++					++++++	+++++ +++++								−	Wrammert et al. (39)
	Brazil	IgG ASCs to DENV-3		−	−	+	+	++	+++++++++ +++++++++ ++++	+	−								Garcia-Bates et al. (40)
Mumps	USA (outbreak)	IgG ASCs to mumps virions							++	+++				+				+	Latner et al. (41)
EV-71	Taiwan	IgG ASCs to EV-71 virions		++ +	+++ ++	+++	+++++++++++++++++++				+++						−		Huang et al. (42)
Falciparum malaria^b^	UK (challenge study)		−		−													−	Elias et al. (43)

*Notes: −describes responses measured but absent, +/− describes responses present in a few individuals with the disease, but absent in most; + describes present in the majority of individuals with disease (each + represents 0–100 antigen-specific ASCs, either isotype specific or total as described in “Optimum method”). [+] represents measurement of ALS only (i.e., where enumeration of ASCs is not directly possible). Lines across columns (i.e., __+__) represent data where only a range of days is appropriate for depiction of results. ALS, antibody from lymphocyte supernatant; ASC, antibody-secreting cells; CtxB, Cholera toxin B subunit; PBMCs, peripheral blood mononuclear cells; LPS, lipopolysaccharide; MSHA, mannose-sensitive haemogluttinin; TB, tuberculosis; BCG, Bacille Calmette-Guérin; PPD, purified protein derivative; SapM, secretion of acid phosphatase by *M. tuberculosis*; ETEC, Enterotoxigenic *Escherichia coli*; CFA/II, *E. coli* colonizing factor; CS1, CS3, CS6, coli surface antigens; LT, heat-labile E. coli toxin; LUTI, lower urinary tract infection; PN, pyelonephritis; RSV-F, RSV fusion protein; DENV-3, dengue virus serotype 3; EV-71, Enterovirus-71*.

*^a^Assuming 48 h from challenge to onset of symptoms (see main text)*.

*^b^No data on timing of samples relative to symptom onset [since volunteers treated immediately following detection of parasitemia (twice daily blood samples)], hence details are for post onset of parasitemia (taken to be days 6–7 following challenge; see main text)*.

Antigen-specific ASC responses to the oral attenuated ETEC vaccine in 20 healthy adult volunteers were compared to antigen-specific ASC responses to challenge with oral non-attenuated ETEC in a further 20 healthy adult volunteers in the USA (16). ELISpot to ETEC vaccine or non-attenuated ETEC antigens was used to assay ASC responses at days 7 and 10. This study also compared the utility of assay of ALS to predict ASC responses. Volunteers who received the ETEC vaccine showed an IgA peak response against anti-colonization factor A II at day 7, which was similar to the IgA peak response against anti-heat-labile toxin in volunteers with non-attenuated ETEC infection. ASC responses to the ETEC vaccine and non-attenuated ETEC infection remained elevated between day 7 and day 10. Assay of IgA ALS showed 75–95% sensitivity versus ELISpot (with a twofold increase from baseline defined as a positive result) for both responders to ETEC vaccine and non-attenuated ETEC infection at day 7. For assay of IgA ALS, sensitivity was reduced at day 10 in comparison to day 7.

Antigen-specific ASC responses to OCV (Dukoral) in eight adults were compared to antigen-specific ASC responses in nine adults with culture-confirmed *V. cholerae* O1 diarrheal disease (21). Median peak anti-cholera toxin (CtxB)-specific IgG ASC responses were detectable in both groups at day 7 following vaccination or onset of illness. Vaccinees tended to have lower median anti-CtxB-specific IgG ASCs than patients. Median peak anti-LPS-specific IgG ASC responses were also lower in vaccinees than patients. An IgG ASC response was predominant to CtxB, and an IgA ASC response was predominant to LPS in both vaccinees and patients. Flow cytometry demonstrated that ~50% of antigen-specific ASCs of any isotype expressed markers for gut homing plasmablasts. These cholera-specific plasmablasts may thus form the basis for gut-specific immunity following cholera infection.

In general, antigen-specific ASC responses appeared lower in adults following vaccination versus adults with challenge/natural infection, at least for ETEC and cholera. This might be expected if infection induces coactivation of pattern-recognition receptors or produces a pro-ASC cytokine milieu in comparison to vaccination. One might hypothesize that the antigen-specific ASC response to infection might be over a broader timescale than that in response to vaccination. However, these comparative studies had too few time points to be able to detect this.

#### Gastrointestinal Infections

Other work, primarily from the International Center for Diarrheal Disease Research in Bangladesh, has used ELISpot and assay of ALS to further define the antigen-specific ASC response in cohorts of patients with ETEC, cholera, typhoid, and tuberculosis infections.

The antigen-specific ASC response was assessed in 46 adult patients with ETEC infection and 10 adults and 10 child healthy controls in Bangladesh (17). An IgA ASC response against colonization factor CS6 could be detected as early as 2 days following onset of diarrhea and then increased 30-fold by day 7. Healthy controls had no detectable antigen-specific IgA ASCs. In comparison, CS6-specific IgG ASCs were also detectable at day 2, increased sixfold at day 7, and were undetectable in healthy controls. All patients with ETEC infection showed IgA ASC responses, but only 75% of patients showed IgG ASC responses. Compared to ELISpot, assay of ALS to both Ig isotypes showed that IgA ALS had a sensitivity of 89% for detection of antigen-specific IgA ASC, and IgG ALS had a sensitivity of 100% for antigen-specific IgG ASC responses.

The IgA ASC response to cholera infection was assessed in 30 adult patients with cholera infection and 10 healthy adult controls (19). The antigens CtxB, mannose-sensitive hemagglutinin, and lipopolysaccharide (LPS) were used in ELISpot and assay of ALS. Patients showed large and significant increases in mean IgA ASC responses against all three antigens between day 2 and day 7 following onset of infection and were undetectable in healthy controls. ALS responses correlated well with antigen-specific ASC responses for all Ig isotypes. Similar large increases in concentrations of antigen-specific IgA ALS were shown for patients between day 2 and day 7 following onset of symptoms, and undetectable in healthy controls. Both ELISpot for antigen-specific IgA ASCs and IgA ALS were compared to a gold standard of a fourfold rise in anti-vibriocidal antibodies at day 7 or day 21. Antigen-specific IgA ASCs showed a maximum sensitivity of 94% (anti-LPS), and IgA ALS showed a maximum sensitivity of 94% (anti-CtxB ALS). ASCs were undetectable by both ELISpot and assay of ALS in healthy controls chosen from this cholera-endemic region, demonstrating 100% specificity in this setting.

Recent work has showed that children as young as 3 years of age produce antigen-specific IgA ASC, IgA memory B cell, and plasma IgA and IgG vibriocidal antibodies comparable to adults following cholera infection (20). The OCV Dukoral also provides protection from cholera infection in children between 1 and 4 years of age (69). Infants and young children are disproportionately affected by diarrheal disease and pneumonia (discussed below). Both of these are primarily mucosal infections, of which large proportions are vaccine preventable. Further studies of the antigen-specific IgA and IgG ASC response in this age group are therefore needed.

#### Enteric Fever (Typhoid and Paratyphoid Infection)

Infection with *Salmonella enterica* serovar Typhi or Paratyphi occurs *via* the faeco-oral route. Unlike cholera, invasion beyond the intestinal mucosa may cause a systemic illness referred to as enteric fever. The antigen-specific ASC response to typhoid/paratyphoid is of considerable interest for the development of diagnostic tests with increased sensitivity in comparison to culture of blood (22, 23). Typhoid-specific ASC responses may also inform the development of new typhoid vaccines (70).

Antigen-specific ASC responses were assessed in 112 adult patients in Bangladesh with suspected enteric fever and 3–7 days of fever at presentation to hospital. ALS assay was done at presentation, day 5 and day 20, against the typhoid antigens LPS, whole-cell preparation, and membrane preparation (MP) (22). Patients were classified into comparator standards for their *a priori* likelihood of true typhoid infection upon the basis of blood culture results, serum Widal titers, and clinical features, with the addition of a healthy control group. A positive (≥2 standard deviations above the mean of healthy controls) anti-MP IgA ALS was detected in all patients with blood culture-positive typhoid infection. Mean IgA (and IgG) ALS responses at day 5 increased across comparator standards of blood culture-negative patients as the likelihood of true typhoid infection increased. Mean ALS responses had returned to near baseline by day 20.

A further cohort of 243 patients in Bangladesh from 1 to 59 years of age with suspected enteric fever and 3–7 days of fever were assessed for ASC responses with similar ALS assay methods and time points (23). Similarly, patients were classified into comparator standards for their *a priori* likelihood of true typhoid infection, with the addition of age-matched healthy controls (74 people). As previously, all patients with blood culture-positive typhoid (or paratyphoid) had positive anti-MP IgA ALS responses. Healthy controls all had negative ALS responses. The proportion of patients with positive ALS responses increased as the likelihood of true enteric fever increased across comparator standards. Of the 38 patients with positive anti-MP IgA ALS response, 32 (84%) had converted to a negative response by day 21 following enrollment (days 24–28 of illness). Five of the remaining six patients continued to be bacteremic for typhoid.

Taken together, the data from these cohorts suggest that the assay of anti-MP IgA ALS responses is a sensitive test for the diagnosis of enteric fever. In the absence of a single gold standard, the increasing proportion of positive ALS responses as the likelihood of enteric fever increased in comparator standards suggests that assay of ALS may also be specific for enteric fever.

#### Tuberculosis

Tuberculosis is typically a chronic intracellular infection due to *Mycobacterium tuberculosis*. Protective immune responses against tuberculosis are generally thought to be mainly T cell-mediated (71). A B cell response with anti-tuberculin IgG may prevent reactivation following containment within tuberculous granulomas (72–74), suggesting that both ASCs and other B cell functions are also required for protective immunity. Despite its global importance, vaccination against tuberculosis using Bacille Calmette-Guérin (BCG) has poor efficacy against pulmonary tuberculosis (the most common clinical manifestation). Tuberculosis remains difficult to diagnose, particularly in those living with HIV infection, the malnourished, and young children. The use of the antigen-specific ASC response to tuberculosis to assess novel vaccines and for diagnosis of disease is thus of considerable interest.

Forty-nine adult patients with sputum smear-positive active pulmonary tuberculosis in Bangladesh were assessed by assay of IgG ALS to BCG antigens (24). Patients with active tuberculosis had mean BCG-specific IgG responses that were almost an order of magnitude higher than the comparator standards of patients with other lung diseases (35 patients, with bronchiectasis, lung cancer, lung abscess, and aspergillosis) and healthy controls (35 BCG vaccinated adults). Optimization of the ELISA threshold value for positive ALS responses gave a sensitivity of 92% and specificity of 80%. These findings were validated in a further cohort of 212 adult patients with suspicion of pulmonary tuberculosis and classes of patients with other lung infections and healthy controls as comparator standards (26). Here, numerous *M. tuberculosis* antigens were assessed for the detection of anti-tuberculosis IgG ALS in ELISAs. Using a threshold value similar to that of the previous study, assay of BCG-specific IgG ALS gave a sensitivity of 90% and specificity of 88% for the diagnosis of active tuberculosis.

Assay of ALS has also been studied for the diagnosis of active pulmonary tuberculosis in children in Bangladesh (25). In this study, children with chronic febrile illnesses aged 11 months to 15 years of age were classified as tuberculosis patients (58 patients) or non-tuberculosis patients (16 patients, in whom tuberculosis was initially considered as a differential diagnosis, but ultimately diagnosed with Hodgkin’s lymphoma, intestinal carcinoma, non-specific lymphadenitis, pneumonia, and pneumonitis). Additionally, 58 age-matched healthy control children were enrolled. BCG was used as the antigen for the detection of anti-tuberculosis ALS. Using an ELISA threshold value similar to that of adult studies, the sensitivity of assay of IgG ALS to BCG antigens at baseline was 91%. In comparison, standard clinical scoring charts over 6 months of follow-up had a sensitivity of 68%. All non-tuberculosis patients and healthy control children had negative ALS responses, suggesting a very high specificity of the ALS assay. Anti-BCG IgG ALS titers had reduced significantly by days 60 and 180 in patients with (now treated) tuberculosis, with the median response less than the positive cutoff value.

A more recent study (28), again from Bangladesh, assessed the use of assay of ALS to BCG antigens for the diagnosis tuberculosis in 224 children admitted to hospital with radiological features of pneumonia and acute severe malnutrition. In this group of patients, using a similar ELISA threshold, sensitivity of assay of ALS was 67% and specificity 45% for the diagnosis of tuberculosis. Time points for blood sampling were not reported. It is unclear whether the relatively unselected nature of this cohort, the timing of sampling relative to treatment, or a reduction in immunity secondary to chronic malnutrition, reduced sensitivity and specificity of assay of ALS in this cohort in comparison to previous studies.

In addition to assay of ALS, ELISpot may also be useful for the diagnosis of tuberculosis. ELISpot was used to enumerate IgA ASCs and memory B cells from the blood of patients with active tuberculosis, adults with latent tuberculosis, and healthy control adults in Uganda (45). Four tuberculosis antigens (ESAT-6, CFP-10, Ag85A, and Ag85b) were used. The most sensitive antigen was ESAT-6. ESAT 6-specific IgG ASCs were twice as high in adults with active tuberculosis compared with latent tuberculosis cases and were undetectable in healthy control adults. *Ex vivo* stimulation of PBMCs showed memory B cells to be more frequent in the blood of individuals with latent tuberculosis than in patients with active tuberculosis, suggesting a role of memory B cells in controlling tuberculosis disease progression. Of 35 healthy control adults, one had an unusual ASC to memory B cell ratio typical of active tuberculosis. Whether this response correlates with a high risk of developing tuberculosis disease is unknown at present.

A comparison of the utility of assay of ALS and ELISpot for the diagnosis of tuberculosis was made in adult patients with sputum smear-negative (but consequently sputum culture positive) tuberculosis, adults with latent tuberculosis and healthy control adults in Ethiopia (27). Assay of ALS to BCG antigens showed 86% sensitivity and 80% specificity in distinguishing patients with active pulmonary tuberculosis from adults with latent tuberculosis. Assay of ALS distinguished patients with active tuberculosis from healthy control adults with 86% sensitivity and 94% specificity. Assay of ALS was considerably more sensitive and specific than the tuberculin skin test and interferon-gamma release assay. Flow cytometry of PBMCs showed that patients with active tuberculosis had higher BCG-specific IgG-secreting plasmablasts than adults with latent tuberculosis or healthy control adults (6 versus 2% of total IgG-secreting plasmablasts).

The data from studies of tuberculosis suggest that the ASC response is readily detectable in patients at presentation to hospital. Assay of BCG-specific IgG ALS appears to have a high sensitivity and specificity in all cohorts assessed, except in a diverse group of malnourished children presenting with pneumonia. ELISpot and flow cytometry have shown that ASCs are detectable directly from blood. The chronic nature of tuberculosis may induce a relatively stable frequency of ASCs in the circulation. This may facilitate the detection of the tuberculosis-specific ASC response at different time points in patients with tuberculosis disease.

#### Pneumococcus and Other Bacterial Respiratory Tract Infections

Colonization with *Streptococcus pneumoniae*, usually in the nasopharynx, is common in children and a prerequisite for pneumococcal disease, but most children colonized do not develop disease (60). Protective immune responses against pneumococci thus involve both prevention of infection (through clearance of nasopharyngeal colonization) and response to infection (should the mucosal surfaces be overwhelmed).

Infants and young children are likely to become colonized with pneumococci on first exposure to a serotype. Salivary IgA against pneumococcal pili proteins is secreted by ASCs in adenoids isolated from healthy children (75). Low antigen-specific salivary IgA from adenoids and serum IgG is associated with pneumococcal colonization, suggesting a protective role for mucosal antibody derived from local ASCs (76). Adults produce a significant increase in anti-capsular pneumococcal IgG to pneumococcal serotypes in response to pneumococcal colonization (77). The extent to which these responses to colonization are detectable as changes in populations of pneumococcus-specific ASCs in peripheral blood in adults or children is unknown.

Pneumococcal pneumonia represents failure to contain pneumococci to the mucosal surface leading to widespread mucosal (alveolar) disease and in some cases bacteremia. A cohort of 16 adult patients in Finland with bacteremic pneumococcal pneumonia all had detectable pneumococcal-specific IgG ASCs in peripheral blood on day 7 following detection of bacteremia (30). For each patient with pneumonia, the ELISpot antigen was the killed invasive pneumococcal isolate detected from the blood of the patient. In all patients, pneumococcal-specific IgG ASCs could be detected. In one of these patients with disease caused by serotype 14, there was also an ELISpot response against a mixture of purified capsular polysaccharides (3, 4, 5, 6B, 7F, 8, 14, 19F, and 23F). IgM and IgA ASCs were detected at lower frequencies. In healthy controls (14 adults), the ELISpot antigens were either the same mix of capsular polysaccharide antigens (*n* = 8) and/or killed pneumococcal isolates (*n* = 14). None of the healthy controls showed IgG responses against any of the pneumococcal antigens. Total ASCs (i.e. not only pneumococcal-specific) were 10 times higher in cases than in controls, suggesting a significant polyclonal ASC response.

Pneumococcal-specific ASCs derived from patients with bacteremic pneumococcal pneumonia (*n* = 16, from the study described above) express a unique set of homing receptors in comparison to pneumococcal-specific ASCs following pneumococcal vaccination (*n* = 14 with pneumococcal polysaccharide vaccination and *n* = 11 with pneumococcal conjugate vaccination) (78). The authors suggest that pneumococcal-specific ASCs may therefore be derived from, and home to, the lung mucosa. Work by the same group has recently characterized the antigen-specific ASC response to tonsillitis and sinusitis in 24 adults caused by *H. influenzae, S. pneumoniae, S. pyogenes*, or other β-hemolytic streptococci (31). Peripheral blood samples were taken 7–14 days following onset of symptoms. These patients all showed detectable IgG ASC responses specific to the causative pathogen, with slightly higher ASC frequencies in sinusitis than tonsillitis.

Taken as a whole, pneumococcal disease induces antigen-specific IgG ASC responses that are detectable in the peripheral blood of adults during illness. These ASC responses are present with or without detectable bacteremia. *H. influenzae* and non-pneumococcal streptococcal respiratory mucosal infections induce a similar immune response.

#### Urinary Tract Infection (UTI)

Like bacterial pneumonia, UTI is usually a bacterial mucosal infection that may rarely invade to cause bacteremia. However, with appropriate sampling techniques, culture of urine is sensitive and specific for disease etiology in contrast to culture of blood or nasopharyngeal specimens in bacterial pneumonia.

A study of the antigen-specific ASC response to UTI in adults caused by *E. coli* and other bacterial pathogens found that 10/14 (71%) patients with lower UTI and 17/17 (100%) patients with pyelonephritis had responses to a preparation of the cultured and killed *E. coli* isolate detectable by ELISpot at 7–8 days following onset of symptoms (32). In pyelonephritis, the mean antigen-specific ASC frequency was sevenfold higher for IgA than IgG. Subsequent studies in adults have confirmed that IgA ASCs predominate in UTI (rather than IgG as in pneumococcal pneumonia), and that ASCs specific for the virulence factor P fimbria are more frequent in *E. coli* pyelonephritis than uncomplicated lower UTI (33). This has biological plausibility, since P fimbriated *E. coli* has increased uroepithelial binding and are associated with increased virulence in UTI (79). Following activation in the urinary tract, antigen-specific ASCs express a unique set of homing receptors that may induce migration to regional lymph nodes (34).

A further study of the antigen-specific ASC response to *E. coli* pyelonephritis in children found a number of differences between the adult and child immune response (80). Here, 33/37 (89%) children aged 1 month to 16 years had responses detectable by ELISpot at either admission or 7 days following admission to hospital. Among all ages, mean antigen-specific ASC frequencies were comparable for IgA and IgM but lower for IgG. Antigen-specific ASC frequency was positively correlated with increasing age, and all four non-responders were infants less than 1 year of age. In contrast to adults, among antigen-specific ASCs, IgM ASCs predominated in children less than 2 years of age, while IgA ASCs predominated in children more than 2 years of age. This suggests that either younger children have a reduced ability to class-switch to IgA ASCs in response to UTI or that a systemic IgM ASC response to UTI is more important in younger children than in older children. Alternatively, older children may have previously been colonized in the urinary tract with *E. coli* and have a regional memory B cell population primed to secrete IgA. The nuanced differences between younger children and older children and adults will have significant implications for the detection of the ASC response to infection (see “Variation in the B cell response between individuals” section).

### Antigen-Specific B Cell Responses to Viral Antigens

#### Influenza

Infection with influenza A and B viruses cause moderate upper respiratory tract or gastrointestinal symptoms in most adults but can cause life-threatening pneumonia in individuals of all ages, but particularly at the extremes of age and pregnant women (81). ItsRNA genome evolves rapidly from year to year through a process of antigenic drift, with occasional reassortment of hemagglutinin (H) and neuraminidase (N) antigens, leading to pandemics of novel influenza viruses (most recently the H1N1 strain emerging in 2009) (82).

Human challenge studies have provided data on the immune response to respiratory infection with influenza virus (35, 83, 84). In most adults, challenge with influenza virus leads to rapid viral replication over 2–3 days during which the host is highly infectious. The majority of adults develop symptomatic disease by 2 days following challenge, with symptoms peaking at day 4 following exposure. Viral replication, as measured by viral shedding, is rare beyond 5 days following challenge (approximately 3 days following onset of symptoms) as a result of the induction of innate and adaptive immune responses.

In a nasal influenza challenge study in 12 adults, blood samples were taken at day 2, day 3, day 7, and day 28 relative to challenge (35). PBMCs were sorted for plasmablast markers before influenza-specific IgG ASC frequency was assessed in these cells using ELISpot. Influenza-specific ASCs peaked in peripheral blood at day 7, and were absent at day 2, day 3, and day 28 relative to challenge. Frequency of influenza-specific IgG ASCs in the peripheral blood positively correlated with peak viral load, length of viral shedding, and symptom severity. In summary, data from adult influenza challenge studies of a show the emergence of influenza-specific ASCs between day 3 and day 7 following challenge, as detected by ELISpot.

Analysis of the transcriptome has been used to investigate gene-level changes in response to influenza challenge. In 17 adult participants, the transcriptome was measured at 8–24 hourly intervals for 96 h (4 days) following challenge (84). Strong immune responses at the interface between innate and adaptive immunity (for example, the toll-like receptor pathway) were detected at 48 h, approximately 36 h before peak symptom time. Notably, the upregulation of two genes was able to accurately classify symptomatic from asymptomatic subjects: FGF9 (fibroblast growth factor 9, implicated in lung epithelial repair) and TLN1 (Talin-1, a cytoskeletal protein). Talin-1 is essential for the migration of leukocytes to sites of inflammation, or to lymph nodes, and also stabilizes interactions between leukocytes (such as *via* the TCR) (85). However, RNA transcripts related to ASC activation and antibody-secretion were not detected within the first 96 h. This is consistent with the absence of influenza-specific ASCs until after at least day 3. Alternatively, hypervariable transcripts (such as those encoding immunoglobulins) may be missed by the use of microarray technology. A more sensitive approach for the detection of transcripts relating to hypervariable immunoglobulin/BCR genes is described below.

The evolving repertoire of plasmablast BCRs (and secreted antibodies) was studied in nine adult patients with moderate to severe pandemic H1N1/2009 influenza infection (86). Only a single time point (typically day 10 following onset of symptoms) was used for blood sampling. Here, flow cytometry and ELISpot demonstrated the presence of influenza-specific IgG plasmablasts, which were absent in unvaccinated healthy volunteers. Single cell capture, PCR amplification and sequencing of the heavy Ig locus (*IGH*) and light Ig (*IGL/IGK*) loci from these plasmablasts showed a highly somatically mutated repertoire of BCRs. High somatic hypermutation may be secondary to the derivation of plasmablasts from memory B cells through a recall response. However, in the context of the early pandemic of influenza H1N1/2009, it is likely that a proportion of the somatic hypermutation represents rapid molecular evolution of plasmablast BCRs occurring over the first 10 days of infection. This has significant implications for the hypothetical utility of ELISpot or assay of ALS for the diagnosis of the etiology of respiratory infections. For example, delaying sampling until several days following onset of symptoms might increase sensitivity (due to increased plasmablast numbers) and specificity (due to increasing affinity of BCRs to influenza antigens).

#### Respiratory Syncytial Virus (RSV)

Respiratory syncytial virus is a major global pathogen causing upper and lower respiratory tract infection in young children and causes repeated symptomatic disease in otherwise healthy adults (87). Like influenza, RSV is an RNA virus, predisposing it to rapid evolution under selective pressure due to the inherent error rate when copying the single-stranded RNA genome. As with influenza virus infection, infection-derived immunity to RSV is of short duration (36).

In a nasal RSV challenge study in 61 adult volunteers, blood was sampled at days 0, 3, 7, 10, 14, and 28 post-inoculation (36). Using flow cytometry and ELISpot, IgA and IgG ASCs specific to RSV lysate were detectable at day 10 following challenge and undetectable before day 7 and after day 14.

In a study of 40 adults with natural RSV infection, blood was sampled at admission, days 10–16 following admission and again in convalescence (days 22–45) (37). RSV was detected in the nasopharynx of all patients, and patients shed RSV for a mean of 11.9 days (range 4–30 days). RSV fusion (F)-protein antigen was used for ELISpot. RSV-specific IgG ASC responses were detected using ELISpot in 36/40 (90%) patients within 11 days following onset of symptoms. RSV-specific IgG ASCs were detectable by day 2 and remained detectable until at least day 12. These ASCs were not detected in patients with other respiratory tract infections. However, 7/9 (78%) participants with a second sample at 8–16 days remained positive by ELISpot (>4 standard deviations above the mean for previously studied healthy controls). Of participants with a second sample at 22–45 days, 11/23 (48%) remained positive by ELISpot. Overall, 16/36 (44%) participants had positive responses beyond the last day of RSV shedding. Thus, RSV infection appears to result in antigen shedding of approximately 12 days, with ASC responses prolonged beyond this in many individuals.

A further adult study involving 97 participants assessed the frequency of antigen-specific ASCs during RSV and influenza infection (*n* = 11 each) as well as in healthy controls including 19 adults recently vaccinated with trivalent influenza vaccine, tetanus toxoid vaccine, human papillomavirus vaccine (HPV) and hepatitis B vaccine (38). Only a single blood sample was taken at enrollment. All RSV-infected patients had RSV-specific IgG ASCs responses detectable with ELISpot at days 2–11 following symptom onset. RSV-specific IgG ASCs were not detected in patients with influenza virus infection or in healthy controls with and without recent vaccinations. Similarly, influenza-specific ASCs were not detected in patients with RSV infection (except in a single-coinfected individual). The specificity of the ASC response to either RSV infection, or influenza infection suggests that ELISpot may be a useful test to distinguish respiratory viral infection from nasopharyngeal carriage. These methods may also be useful to investigate the immune response to RSV with the aim of developing an effective vaccine against this pathogen.

#### Dengue

Dengue virus consists of four serotypes that are transmitted by *Aedes* spp. mosquitoes in the global tropics (88). Most dengue virus infections are asymptomatic or cause mild febrile illness. More rarely, infections can cause life-threatening disease characterized by vascular endothelial damage, cardiovascular shock, and rapid consumption of platelets leading to coagulopathy (severe dengue) (89).

Severe dengue infection appears to be associated with high plasmablast frequencies *in vivo*. In a cohort of 46 adult patients admitted to hospital with severe dengue infection in Bangkok, dengue-specific IgG plasmablasts were identified by flow cytometry combined with ELISpot (39). These plasmablasts were detectable at levels above that of healthy controls from day 4 following onset of symptoms, and peaked in frequency between day 6 and day 7. Median frequency of dengue-specific IgG plasmablasts was ~10,000 per million PBMCs, representing at least a 1,000-fold increase from healthy adult controls. These plasmablast frequencies are considerably higher than that displayed in other severe viral (and bacterial) infections (90, 91). A further study in South America has confirmed this rapid and massive plasmablast response and found peak plasmablast frequency to be associated with disease severity (40).

In a prospective study of 28 adult patients with severe dengue, the transcriptome showed enrichment for monocyte and macrophage-associated genes in comparison to controls (92). Enrichment for these genes was associated with higher viremia. Monocytes infected with dengue virus secreted proinflammatory cytokines such as interleukin 1 receptor antagonist and induced the differentiation of B cells into plasmablasts *in vitro* after 6 days. Similar findings have been noted in other cohorts of children and adults (93, 94). Identification of dengue-specific plasmablasts (or their secretions) with ELISpot or ALS may represent a sensitive and specific diagnostic test for dengue infection from day 4 of febrile illness. This may be especially relevant in endemic regions where seropositivity is common in healthy children and adults.

The observations that (a) severe dengue occurs during rapid clearance of the virus, rather than during maximum viremia, (b) severe dengue mainly occurs in secondary infection with a heterotypic dengue virus serotype, and (c) plasmablasts are positively correlated with severity of infection, implicates the host immune response in the pathogenesis of severe dengue (95). Current evidence supports the theory of antibody-dependent enhancement of disease (96): invasion of dengue virions into monocytes (and possibly B cells) through the FCγ receptor is augmented by weakly reactive B memory cell derived antibodies to protein M on the viral surface (97, 98). These virions are rendered hypoimmunogenic (99), resulting in rapid intracellular replication and leading to high viremia in patients with severe dengue disease. Whether the phenotype of severe dengue is caused by the humoral immune response, or by a correlated T cell specific response is undetermined (100). Nonetheless, concerns regarding this phenomenon have hampered the development of a vaccine to dengue virus, further emphasizing the importance of pathogen-specific studies of the B cell response to infections.

#### Other Viral Infections: Mumps, Enterovirus

Infection with mumps virus causes parotid gland swelling and a moderate febrile illness in the majority of individuals, but may cause meningitis and encephalitis, and oophoritis, orchiditis in girls and boys, respectively. Complications include deafness and sterility (101). Mumps virus infection, or two or more live attenuated vaccinations (typically with measles-mumps-rubella vaccination, MMR), produces long-lasting immunity (102). However, prior vaccination complicates serological diagnosis of mumps infection, and assay of the ASC response to infection with either ELISpot or ALS may be useful in an outbreak situation.

ELISpot was used to assay mumps-specific IgG ASCs in an outbreak of mumps infection in seven previously vaccinated adults in the USA. Blood samples were taken at a single time point between day 7 and day 28 following onset of symptoms. All seven patients had detectable mumps-specific IgG ASCs significantly above a threshold determined from studies of vaccinated individuals. The single patient sampled at day 28 had a mumps-specific IgG ASC count that was just above the threshold. Assay of antigen-specific ASCs may thus be a useful diagnostic test in the context of prior vaccination. Further investigation of the BCR or antibodies derived from these ASCs might also illuminate the underlying reasons for vaccine failure in these individuals.

Enterovirus-71 (EV-71) is an RNA virus that has caused epidemics of herpangina and has been implicated in epidemics of life-threatening encephalitis and multiple organ failure in children in the Asia-Pacific region (103). Biennial epidemics of EV-71 infection occur, mainly in children aged 1–3 years. These epidemiological patterns, similar to pre-vaccine era measles epidemics (104), suggest a protective role for humoral immunity, with epidemics following the accrual of sufficient unprotected infants to allow widespread transmission.

The ASC response to acute EV-71 infection was assayed using ELISpot and flow cytometry on blood from 28 children with laboratory confirmed genotype B EV-71 infection. Blood was sampled once between day 2 and day 11 following symptom onset, and once in convalescence (>19 days following symptom onset) for each child. Acute blood samples showed EV-71-specific IgG and IgM ASCs and lower IgA ASCs. Analysis of ASCs of all Ig isotypes showed a strong ASC antigen-specific response at days 1–3, which further increased ~5-fold at days 4–7, and was almost absent from day 8 onward. Children aged <3 years had a population of EV-71-specific ASCs that almost entirely secreted IgM, while children aged >3 years showed a predominantly IgG ASC response. Enterovirus-specific IgG ASCs may either represent rapid class-switching in older children, or recall memory.

Thus, the ASC response to EV-71 infection in children has a similar kinetic to that described for influenza, RSV, dengue virus, and mumps virus infections. This suggests that there is either a conserved pathway to ASC activation and proliferation or several redundant pathways converging to the same endpoint.

### Protozoa

#### Falciparum Malaria

Clinical malaria results from infection with one of four human *Plasmodium* spp. protozoa, with the majority of severe disease (anemia, acidosis and cerebral malaria) caused by *Plasmodium falciparum* infection. Naturally acquired immunity to falciparum malaria is generally slowly acquired (amongst survivors) over the course of repeated infections in childhood in areas of endemic transmission (105). Following inoculation, initial invasion of the bloodstream by pre-hepatic sporozoites of *P. falciparum* does not induce a protective humoral response (106). Only with onset of clinical malaria following dissemination of merozoites is a protective humoral response induced.

In 38 children approximately 5 years of age with clinical malaria in Uganda, plasmablasts were assayed by flow cytometry over 28 days following presentation (107). Plasmablasts were already at peak levels (~6% of peripheral blood B cells) at time of presentation to hospital. These declined by day 7 to ~3% of peripheral blood B cells following treatment for malaria (107). In contrast, memory B cells remained at ~10% of peripheral blood B cells over 28 days. Atypical memory B cells (hypothesized to represent “exhausted” non-function B cells (108)) increased from 10 to 16% of peripheral blood B cells and were positively associated with parasitemia on presentation. However, this study did not estimate the malaria-specific proportion of ASCs (or memory B cells), limiting its generalizability to the studies of the ASC response to infection.

The difficulty of defining onset of malaria infection in areas of endemic transmission (where a large proportion of children may have asymptomatic parasitemia) makes data from malaria challenge studies important. The majority of studies reporting B cell responses to malaria infection have focused on memory B cells and often in the context of malaria vaccination strategies. However, one study quantified both the acutely activated malaria-specific ASC response following vaccination with merozoite surface protein 1 (MSP1) and the ASC response following malaria challenge infection. In this study, 9 healthy adults were enrolled and participated in a phase II trial of MSP1-based vaccination, with blood samples taken at days 5, 7, 8, 9, and 15 following booster vaccination. Immediately following day 15, 8 of 9 volunteers subsequently underwent malaria challenge infection with samples at baseline (day 16 following booster vaccination), day 7, day 11 (4 volunteers), and day 35 following challenge. ELISpot to the MSP1 and apical membrane 1 antigen (AMA1) was used to enumerate ASCs.

In these nine volunteers, MSP1-specific ASC responses post-vaccination were similar in timing (peak at day 8 and absent at day 15) and magnitude to those described for bacterial and viral pathogens above. However, following malaria challenge infection MSP1-specific ASCs were undetectable at all time points measured. This intriguing result suggests that either IgG ASCs induced by malaria challenge are not cognate for MSP1 (unlike ASCs induced by vaccination, and despite this being a dominant antigen for inducing IgG memory B cell responses), or that malaria infection downregulates the normal ASC response to infection, or that the timing of the malaria-specific ASC response differs to that of other acute infections. Given that only blood-stage merozoites induce a significant protective humoral response, and that merozoites emerged at approximately day 7 following challenge, one might expect peak malaria-specific IgG ASC responses to be identified at days 14–17 following challenge (i.e. days 7–10 following emergence of merozoites).

In summary, the ASC response to malaria infection (with an apparent decrease in ASCs during clinical infection) is unlike the responses to bacterial and viral infections (where large increases in ASCs are typical). In studies of natural infection, this phenomenon may be an artifact of delayed presentation of children to healthcare facilities; or the comparatively long time from infection to clinical [approximately 10–14 days from inoculation (105)]. The results of malaria challenge infection suggest, however, that malaria may induce a maladaptive ASC (and possibly B memory cell) response through an unknown mechanism. This would be also in accord with the observed slow generation of immunity to clinical malaria and the loss of immunity to malaria in individuals who migrate out of malaria endemic regions.

## Future Study and Clinical Applications

### Variation in the B Cell Response between Individuals

Despite the conservation of the ASC response to infection across pathogens (Table 2), a small proportion of individuals do not appear to produce the “normal” ASC response. Rather, some individuals have low or undetectable ASC responses even in confirmed infection. Quite why some individuals exhibit an abnormally low ASC response to the antigens under investigation is unknown. One possibility is that testing only 1–4 antigens in ELISpot or ALS is simply insufficient, and some individuals have an immune response that targets untested pathogen antigens. Such a possibility could be investigated using ALS against a protein array derived from pathogens of interest.

Alternatively, it may be that genetic variation in lymphocytes (109), or in the regulation of the innate and immune response (59), is fundamental in the ASC response to T cell dependent and independent antigens (110, 111). Stochastic mechanisms probably determine B cell fate at the level of the individual cell (4, 112), which may be of importance in B cell memory (where the small number of memory B cells leads to a small repertoire of antibodies/BCRs in the quiescent state). However, given the large number of activated cells, this is unlikely to be of importance in acute infection. Immunological diagnostic tests may also be of limited use in the important group of patients with an impaired immune response (113, 114).

The relevance of abnormally low or high ASC responses to clinical outcome is unclear. As we have described, worsening clinical status in dengue virus infection is correlated with increasing frequency of dengue-specific plasmablasts (40). It may be that plasmablast-mediated pathology is an under-appreciated aspect of a number of diseases. Generally, however, one would expect that lower ASC responses and a hypo-immune state, would correlate with a worse clinical outcome. An immunosuppressed phenotype does appear to correlate with mortality in severe sepsis (53), but to what extent this correlates with changes in B cell and T cell compartments and to what extent this changes during evolving sepsis (115) are topics for future study.

### Future Clinical Applications of B Cell Responses to Infection

#### The Use of ASC Kinetics for the Diagnosis of Infections

In this review, we have detailed the application of ELISpot and ALS for the study of the ASC response to infection (Table 1). Many of these studies have been done with the goal of developing novel diagnostic tests for a specific pathogen and in patient groups where there is a gold standard against which the results can be compared (e.g. RSV and influenza virus infection). Further assessment of the assay of ASC responses as diagnostic tests for specific pathogens needs to be done. For example, optimizing the antigens to use for detection of ASCs and defining the optimum isotype of BCR for each infection (which may also differ according to the age of child). Most data indicate that the ASC response peaks at approximately 7 days following onset of illness (with the exception of malaria). However, future studies should consider using more frequent time points (e.g. daily samples for duration of hospital admission) in order to provide important detail on the timing of the ASC response.

Assay of antigen-specific ASCs for the diagnosis of the etiology of infection in prospectively enrolled case series is an important future challenge. Pneumonia provides a prototypical example, with multiple potential etiologies. Initial results from the Pneumonia Etiology Research for Child Health (PERCH) project suggest that microbiological diagnostic tests may be insensitive for bacteria (116). Studies of the impact of vaccination against specific pathogens (“vaccine probe studies”), nested within randomized controlled trials, provide epidemiological evidence for the etiology of pneumonia (117–119). Such studies rely on the availability of new vaccines. Alternative paradigms of infectious disease diagnostic testing, based on the highly specific ASC response to infection, or potentially the host transcriptome response to infection (51), are therefore attractive areas of future research.

The data presented here show the ASC response to differ greatly between individuals with similar or identical pathogen stimuli. Variability in the host immune response to infection, particularly with regard to genotype (58) and immune regulation during infection (59), remains an important challenge, with implications both for developing diagnostics based on the immune response and for understanding disease risk associated with this variability. Additionally, some pathogens, such as malaria, Epstein–Barr virus, and others, subvert the B cell-mediated response, resulting in chronic infections (120). Alternatively, pathogens such as dengue virus appear to augment the numerical ASC response to infection, resulting in severe immunopathology (95). Other pathogens, such as *Staphylococcus aureus*, induce a potent humoral response against secretory proteins thereby biasing the response away from replicating bacteria (121). A detailed understanding therefore of both the heterogeneity of the immune response to a given infection and the varying strategies of pathogens to evade this response will inform the development of diagnostic testing. Such data will also be highly relevant to the development of vaccines and therapeutic monoclonal antibodies.

#### The BCR Repertoire to Investigate the Etiology of Infections

High-throughput genetic sequencing of the *IGH* locus and *IGL* and *IGK* loci that encode the BCR is now feasible allowing millions of BCR sequences to be determined simultaneously from a single sample (122). Technical challenges remain, including linking *IGH* and *IGL/IGK* sequences to represent the BCR on a single ASC and distinguishing genuine hypervariability in transcripts from read-error. Bioinformatic methods must also qualify the constraints on the affinity maturation of the BCR repertoire due to the baseline diversity of BCR sequences [so-called “original antigenic sin” (123)]. However, current data show that, following vaccination with either polysaccharide or protein–polysaccharide conjugate antigens, the detection of BCR transcripts can be used to detect ASCs in PBMCs in an unbiased manner. Further, the plasmablast BCR repertoire undergoes convergent immunological selection to a small number of vaccine epitopes, suggesting that antigen-specific ASCs can be detected (56, 57) The incremental development of a library of sequences that represent BCRs cognate to a variety of vaccine epitopes is therefore significant (54, 124).

If a similar process of convergent selection occurs during infection, then detection of pathogen-specific BCR sequences could define humoral immunity to different pathogen antigens at a fundamental genetic level (55, 122). Such an approach could be used for the etiological diagnosis of infection in a (theoretically) unbiased manner. A further theoretical advantage is an increased sensitivity early in the time course of infection due to PCR amplification of the BCR repertoire (based on primers to conserved regions of the BCR sequences). The successful application of this technique may therefore define etiology of infection in a patient cohort or to develop therapeutic monoclonal antibodies to existing, or emerging pathogens (125, 126).

#### Therapeutic Utility of B Cell Studies: Monoclonal Antibodies

Monoclonal antibodies may become important in the treatment of infectious, particularly in the context of increasing antimicrobial resistance and emerging infectious diseases. For example, the identification of a monoclonal antibody (FI6v3) that is capable of neutralizing all known human influenza isolates has clear therapeutic potential for patients with severe influenza. This was, however, time-consuming and derived from the screening of over 100,000 plasmablasts (127). Recent advances in enriching antigen-specific plasmablasts from vaccinees is likely to considerably reduce the time and expense required to identify broadly neutralizing antibodies (128).

Identification of broadly neutralizing antibodies to HIV from individuals who are long-term non-progressors to AIDS provides a novel method to protect against HIV infection. The recombinant gene for the broadly neutralizing antibody is engineered into a viral vector that is optimized for persistent transcription and injected intramuscularly (129). In mice models, long-lasting protection against HIV infection can be achieved. This technique, termed vectored immunoprophylaxis, provides a method to immunize individuals with optimized antibodies. It also does not need an intact immune system and may therefore be particularly useful for “vaccination” against HIV in humans (130).

B cell receptors sequencing may also become an alternative molecular method for identifying broadly neutralizing antibodies to a range of infections. When combined with gene editing techniques such as CRISPR-cas9, this could provide a rapid method to produce monoclonal antibodies against conserved pathogen antigens (125, 131). Theoretically, such an approach may be particularly useful to develop novel therapies for prolonged epidemics of acute infectious diseases such as the Ebola virus epidemic in West Africa of 2014–2016 (126).

## Conclusion

A number of studies have examined the ASC response to infection across diverse bacterial and viral diseases and in malaria. We present a focused analysis on the timing and magnitud, and the clinical applications of these responses. These studies indicate that ELISpot and ALS may be very sensitive and highly specific methods for determining the etiology of infection and have some advantages over current methods.

The timing of the peripheral blood ASC response to infection is conserved for the bacterial pathogens (*V. cholerae, E. coli, S. enterica* var Typhi, *M. tuberculosis, S. pneumonia*, and other bacterial respiratory pathogens) and viral pathogens (influenza viruses, RSV, dengue viruses, mumps virus, and EV-71) for which data are available. Antigen-specific ASCs generally express markers of acute proliferation and are detectable in peripheral blood by approximately day 4 following onset of symptoms, peak in frequency at days 6–8, and are absent from approximately day 11 onward. In contrast to the conserved timing of ASC detection, the magnitude of peak ASC frequency in peripheral blood varies widely between different bacterial and viral pathogens.

At present, data are too few for definitive conclusions. The vagaries of timing of clinical presentation of individuals and the infrequency of blood sampling prohibit a detailed understanding of the ASC response over time (except in a few challenge studies involving a small number of individuals). Variability in the host immune response to infection, particularly with regard to genotype (58) and immune regulation during infection (59) remains an important challenge, with implications both for developing diagnostics based on the immune response and for understanding disease risk associated with this variability. More specifically, details of the ASC response to malaria and other protozoal infections are sparse.

Nonetheless, there is a great potential in the use of antigen-specific ASCs for the etiological diagnosis of infection. Current efforts focus on the broadening and optimization of pathogen detection. This approach, however, is severely hampered by the frequent detection of colonizing organisms and hence difficulties in defining the causative pathogen. Practical further work in this area should include systematic prospective sampling of large number of cases and appropriate controls with a similar clinical phenotype but with a number of potential etiologies. Future studies should also formally integrate data from a variety of current (e.g. clinical features, blood culture) and experimental (e.g. ELISpot, ALS and BCR sequencing) diagnostic methods to estimate the probable etiology of infection in cases. Such data may be useful to assess the burden of disease caused by various pathogens (e.g. in the context of planned vaccine introduction) and ultimately aid the development of rapid diagnostic testing that could inform clinicians working with patients with infectious disease syndromes.

## Author Contributions

MC helped conceive and plan the review and led the writing of the manuscript. RM helped conceive and plan the review and contributed to the writing of the manuscript. PS contributed to the writing of the manuscript. DK helped conceive and plan the review and contributed to the writing of the manuscript. JT helped conceive and plan the review and contributed to the writing of the manuscript.

## Conflict of Interest Statement

The authors declare that the research was conducted in the absence of any commercial or financial relationships that could be construed as a potential conflict of interest.
